# HEE-SegGAN: A holistically-nested edge enhanced GAN for pulmonary nodule segmentation

**DOI:** 10.1371/journal.pone.0328629

**Published:** 2025-08-19

**Authors:** Yong Wang, Seri Mastura Mustaza, Mohammad Syuhaimi Ab-Rahman, Siti Salasiah Mokri

**Affiliations:** 1 Department of Electrical, Electronic and Systems Engineering, Faculty of Engineering and Built Environment, Universiti Kebangsaan Malaysia, Bangi, Selangor, Malaysia; 2 Department of Information and Artificial Intelligence, Wuhu Institute of Technology, Wuhu, Anhui Province, China; Shijiazhuang Tiedao University, CHINA

## Abstract

Accurate segmentation of pulmonary nodules plays a critical role in monitoring disease progression and enabling early lung cancer screening. However, this task remains challenging due to the complex morphological variability of pulmonary nodules in CT images and the limited availability of well-annotated datasets. In this study, we proposed HEE-SegGAN, a holistically-nested edge-enhanced generative adversarial networks, which integrated HED-U-Net with a GAN framework to improve model robustness and edge segmentation accuracy. To incorporate spatial continuity, we constructed pseudo-color CT images by merging three consecutive lung CT slices into the RGB channels. The generator adopted the HED-U-Net, while the discriminator was implemented as a convolutional neural network. Two inverted residual modules were embedded within the HED-U-Net to fuse inter-slice spatial information and enhance salient features using a channel attention mechanism. Furthermore, we exploited the side outputs of HED-U-Net for deep supervision, ensuring that the generated results align with the statistical characteristics of real data. To mitigate mode collapse, we incorporated minibatch discrimination in the discriminator, encouraging diversity in the generated samples. We also improved the loss function to better capture edge-level details and enhance segmentation precision in edge regions. Finally, a series of ablation experiments on the LUNA16 dataset demonstrated the effectiveness of the proposed method. Compared to traditional 3D methods, our approach extracted features more efficiently while preserving spatial information and reducing computational requirements. The use of multi-scale feature maps in HED-U-Net enabled deeply supervised GAN training. The combination of feature matching and minibatch discrimination further improved model stability and segmentation performance. Overall, the proposed pipeline exhibited strong potential for accurate segmentation across a wide range of medical imaging tasks.

## 1. Introduction

In 2022, approximately 20 million new cancer cases were recorded globally, with lung cancer ranking among the top 30 most prevalent types, comprising 12.4% of the total incidence [1]. Studies have shown that timely diagnosis and treatment can reduce lung cancer mortality by up to 20% [2]. Lung cancer often initially manifests as pulmonary nodules [3], making their follow-up monitoring using chest computed tomography (CT) crucial for improving patient survival [4]. However, CT scans consist of hundreds of slices, and identifying pulmonary nodules of varying shapes within a complex anatomical background is a challenging and labor-intensive task [5]. Therefore, the development of accurate and automated pulmonary nodule segmentation algorithms is of great significance for enhancing the effectiveness of lung cancer screening. Unfortunately, accurate lung nodule segmentation presents several challenges. First, the availability of well-annotated data is limited, as clinicians have constrained time for labeling CT images [6]. Furthermore, class imbalance poses a significant challenge in existing datasets. As an example, within the LIDC-IDRI dataset, ground-glass opacity (GGO) nodules make up merely around 6% of cases, whereas most instances consist of either solid or partially solid nodules [7]. Second, pulmonary nodules exhibit diverse morphologies, including solid, cavitary, mixed ground-glass, pure ground-glass, and small nodules, each with distinct locations, contrasts, and textures [8]. Third, the complexity of CT backgrounds, which include effusions, cysts, scars, fibrosis, consolidations, and normal anatomical structures such as blood vessels, can interfere with accurate segmentation [9]. Additionally, the edges of certain nodules, particularly GGO nodules, are often blurred, further complicating their delineation [10]. Moreover, nodules vary significantly in size and occupy only a small portion of the lung image, making segmentation more challenging [11]. With the increasing volume of CT imaging driven by medical advances, processing such data has become both time-consuming and prone to diagnostic error [12].

Due to these challenges, traditional morphological methods struggle to accurately segment all types of pulmonary nodules. Fortunately, the emergence of deep learning has opened new possibilities for precise nodule segmentation, and researchers have made significant progress in this field. Currently, pulmonary nodule segmentation primarily relies on U-Net variants. Nevertheless, the convolution and pooling operations in U-Net can cause spatial information loss, leading to a semantic gap. To mitigate this, researchers have introduced additional network connections and attention mechanisms to preserve semantic information and utilize contextual features. In the study [13], a comparative evaluation of the Sliding Band Filter (SBF), U-Net, and SegU-Net was conducted on the LIDC-IDRI dataset. The results indicated that the deep learning-based U-Net and SegU-Net architectures achieved significantly better segmentation performance than the conventional SBF approach. Wang et al. [14] proposed CLT-Net, which improved segmentation accuracy by strengthening connectivity to fully exploit multi-scale feature maps while integrating an attention mechanism and a boundary-aware loss function. Maqsood et al. [15] proposed an improved U-Net that expanded the receptive field with atrous convolution and enhanced feature extraction through deep connections. Lu et al. [16] proposed DENSE-UNET, which employed dense connection to transfer and utilize features, and alleviate the problem of vanishing gradients. Qian et al. [17] proposed U-Net-sharp, a novel network architecture that enhanced feature connectivity through dense and full-scale skip connections while integrating multi-scale information in the decoder. In addition, the incorporation of deep supervision and classification-guided modules further improved both the accuracy and efficiency of the segmentation process. To better utilize multi-scale features in images, Wang et al. [18] proposed SKV-Net, a lightweight segmentation network based on V-Net. It integrated selective kernel convolutions and soft attention mechanisms to enhance multi-scale feature extraction. While 3D U-Net was initially introduced to better exploit spatial information in CT images, Kido et al. [19] further enhanced it by incorporating deep connections and residual structures, thereby improving feature extraction efficiency and alleviating the vanishing gradient problem. Lin et al. [20] proposed a 3D V-Net network that fully exploited the spatial information in CT images. Although this design improved segmentation accuracy, it comes at the cost of increased model complexity and computational demand. Xu et al. [21] designed a separable 3D convolution to replace the standard convolution in V-Net, reducing computational complexity while enhancing model performance through feature fusion and an attention mechanism.

Some researchers introduced additional modules into U-Net, including multi-scale and residual modules, to increase network depth and enhance segmentation performance. Zhou et al. [22] incorporated residual learning units and an inception structure into the U-Net to capture multi-scale information and mitigate gradient vanishing when deepening the network. Dong and Liu [23] improved U-Net++ by incorporating ResNeXt [24] and squeeze and excitation blocks [25], leading to enhanced receptive fields and more effective feature extraction. Rocha et al. [13] developed Seg-UNet by integrating SegNet’s up-sampling method into U-Net. The architecture leveraged the strengths of both models to restore pixel position information, enhance edge details, reduce computational costs, and improve memory efficiency. Annavarapu et al. [26] proposed a method integrating a bidirectional feature network between the encoder and decoder to enhance multi-scale feature utilization. Tang et al. [27] proposed a feature complementary network based on the pre-trained ResNet-50 backbone to facilitate the mutual fusion and enhancement of diverse features. Moreover, Wu et al. [6] conducted a comparative evaluation of Mask-RCNN, U-Net, SegNet, and DeepLabv3+ on the Lung Nodule Analysis 2016 (LUNA16) and Liver Tumor Segmentation Challenge 2017 datasets, and reported that Mask-RCNN achieved the best performance.

Some researchers explored multi-network collaboration to improve the accuracy of lung nodule segmentation. Zhao et al. [28] proposed a cascaded two-stage U-Net to address the ambiguity between tissue and tumor regions. Wu et al. [12] proposed a dual-branch parallel neural network architecture for coarse-to-fine lung nodule segmentation. The network consists of a 2D branch to extract spatial features from individual slices and a 3D branch to capture inter-slice dependencies. Liu and Pang [29] proposed a lung nodule segmentation method that combined a double dilated U-Net with a multi-scale gray correlation approach. The former was employed to extract lung contours, while the latter improved segmentation accuracy by enhancing gray-level feature associations across scales. Qiu et al. [30] proposed a two-branch complementary model comprising a 3D U-Net for lung nodule region segmentation and an auxiliary branch for edge guidance. A feature fusion module integrated features from both branches to enhance segmentation accuracy.

However, the limited availability of lung nodule data poses a risk of underfitting for overly complex models. To address this challenge, some researchers have explored the use of generative adversarial networks (GANs) for lung nodule segmentation, leveraging their ability to model data distributions and improve adaptability to ambiguous boundaries. Jain [31] proposed a GAN model based on the salp shuffled shepherd optimization algorithm. Tyagi and Talbar [4] developed a 3D conditional GAN incorporating spatial and channel attention mechanisms to learn the probability distribution of pulmonary nodules in CT images, thereby enhancing segmentation accuracy.

While promising, designing and training GANs presents several challenges. It is essential for the generator to accurately learn the probability distribution of real data, and for the generator and discriminator to reach a Nash equilibrium, converging simultaneously. To address the above difficulties, we proposed HEE-SegGAN: a holistically-nested edge enhanced GAN for the pulmonary nodule segmentation. Our main contributions are as follows:

a. To efficiently utilize spatial information in CT images without introducing excessive parameters, we encoded three consecutive slices into the RGB channels, forming a color CT image. Using the LUNA16 dataset, we evaluated U-Net, holistically-nested edge detection U-Net (HED-U-Net), and GAN models. Experimental results demonstrated that the segmentation accuracy of the synthesized images outperforms that of single-slice images.
b. We employed HED-U-Net as the generator in the GAN framework, utilizing its hierarchical feature output to supervise training, capture the real data distribution, and improve stability. Additionally, the hierarchical feature output was integrated with a global attention mechanism to fully exploit multi-scale information in CT images. Two inverted residual modules were designed in HED-U-Net to enhance spatial information fusion and extraction in synthetic CT images.
c. To enhance lung nodule edges, we used pooling to generate multi-scale ground truth and computed loss at different levels in the generator and discriminator. Higher weights were assigned to edge regions to improve segmentation accuracy.
d. We implemented a mini-batch discrimination technique in the discriminator to prevent gradient convergence within the same batch, reducing intra-batch similarity and enabling the GAN to better capture the real data distribution.

## 2. Related work

### 2.1. GAN

GAN [31], first proposed by Goodfellow et al. in 2014, have gained significant attention for their remarkable ability to generate realistic data by learning complex data distributions. Xue et al. [32] proposed SegGAN, which applied GAN to medical image segmentation. Unlike conventional segmentation models, GAN employs a dual-network architecture comprising a generator and a discriminator. In image segmentation tasks, the generator aims to produce segmentation masks that closely approximate the ground truth, while the discriminator assesses their authenticity by differentiating between real and synthesized masks. The discriminator evaluates the segmentation results produced by the generator and provides feedback to guide the iterative learning process. This continuous refinement allows the generator to progressively improve its predictions, thereby enhancing segmentation performance. By combining generative adversarial strategies with advanced feature extraction techniques, GANs effectively preserve fine structural details and improve edge precision, making them well-suited for tasks such as pulmonary nodule segmentation. Furthermore, GAN-based models can be combined with task-specific architectures like HED-U-Net to further enhance performance by incorporating multi-scale and edge-aware features.

### 2.2. HED-U-Net

Xie and Tu [33] proposed the HED-U-Net, which integrated deep supervision to enable multi-level features from different network layers to jointly contribute to the final prediction, thereby enhancing edge detection accuracy. Heidler et al. [34] utilized HED-U-Net for monitoring changes in the Antarctic coastline, leveraging HED to capture fine-grained edge information and improve edge detection accuracy. The HED-U-Net architecture is also applicable to medical image segmentation, particularly in edge-sensitive tasks such as lung nodule and tumor segmentation. Traditional U-Net architectures often struggle to precisely segment objects with complex or ambiguous boundaries, which is particularly challenging in pulmonary nodule segmentation due to the irregular shapes and low contrast of nodules against surrounding tissues. HED-U-Net addresses this challenge by incorporating hierarchical output at multiple scales, enabling the model to capture fine-grained edge details while preserving global structural features. Additionally, the hierarchical outputs can supervise GAN training by guiding the generator to produce data that conforms to the statistical characteristics of real images, thereby enhancing the stability and reliability of the GAN-based segmentation framework.

## 3. Methods

### 3.1. Data preprocessing

In this work, we employed the LUNA16 [35] dataset to validate our algorithm. As a widely recognized benchmark for pulmonary nodule detection and segmentation, LUNA16 is constructed from the LIDC-IDRI dataset. It consists of 888 low-dose thoracic CT scans, each with a slice thickness of at most 2.5 mm. We extracted the content within the range of −600–1200 Hounsfield Units from the original CT image and generated the ground truth based on the point set described in the annotation file. Then, a 128 × 128 region of interest was centered at the second-order moment of the ground truth for extraction.

Lung CT images consist of continuous slices of lung tissue, containing valuable spatial information that aids in nodule diagnosis. Therefore, in pulmonary nodule segmentation, 3D models generally outperform 2D models. However, this improvement often comes at the expense of a substantial increase in model complexity and computational cost. For instance, in the LUNA16 dataset, most CT scans have a slice thickness and spacing of approximately 1 mm. Pulmonary nodules typically range from 3 mm to 15 mm in diameter, implying that a single nodule may span 3–15 consecutive slices. In 3D models, using input volumes such as 64 × 64 × 64 often included many irrelevant slices, resulting in parameter redundancy and decreased training efficiency and segmentation accuracy. To address this issue, and inspired by [36], we adopted a synthetic image approach that effectively leveraged the spatial information in CT scans without introducing excessive parameters.

**Fig 1:**
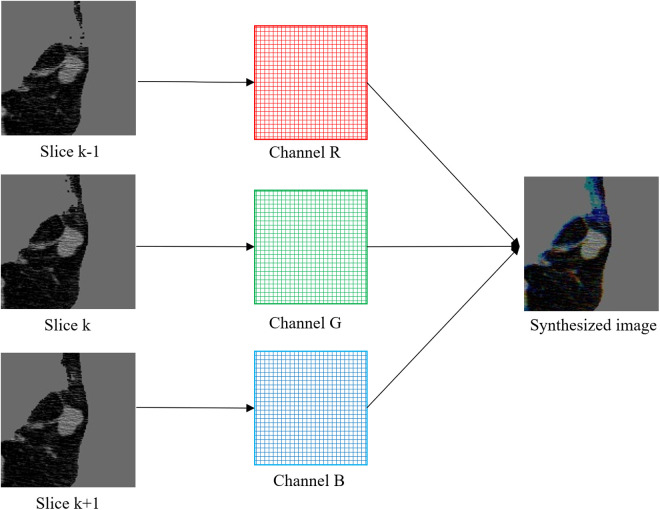
Schematic diagram of image synthesis.

As shown in Fig 1, the CT image is centered on the nodule, and the previous, current, and next slices are encoded into the R, G, and B channels of a three-channel image, forming a color CT image. This approach incorporates information from adjacent slices and preserves spatial context. In clinical practice, radiologists also analyze multiple consecutive slices to differentiate pulmonary nodules from normal lung tissue. This image synthesis method effectively leverages spatial information in CT images. As illustrated in Fig 1, blood vessels appearing across three consecutive slices are colorized, whereas the nodule region, primarily present in a single slice, remains monochrome.

### 3.2. Model framework

**Fig 2:**
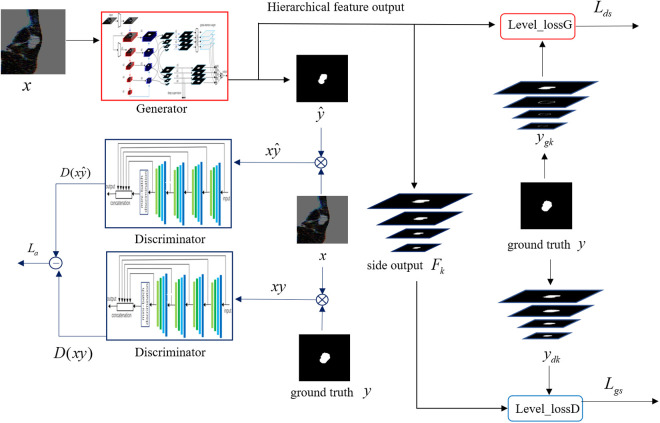
Architecture of HEE-SegGAN.


The framework of HEE-SegGAN is shown in Fig 2. The generator of HEE-SegGAN is a HED-U-Net, while the discriminator is a four-layer convolutional neural network (CNN). Given an input CT image x, the generator produces both the predicted nodule mask $\hat{y}$ and four hierarchical feature maps $F_{k}$, (*k* = 1, 2, 3, 4), which serve as side outputs of HED-U-Net. The side outputs effectively capture multi-scale contextual information across different semantic levels of the network. By multiplying the predicted mask $\hat{y}$ with the original CT image x, the predicted nodule region $x\hat{y}$ is obtained. Similarly, the ground truth mask y is used to extract the real nodule region from the CT image xy. The predicted nodule region $x\hat{y}$ is fed into the discriminator D, producing $D(x\hat{y})$, while the real nodule region xy yielded D(xy). The adversarial loss of the GAN is denoted as $L_{a}$, as defined in Equation (1).

$$L_{a}=\left|D(x\hat{y})-D(xy)\right|$$ (1)

During adversarial training, the generator aims to minimize the adversarial loss $L_{a}$, while the discriminator seeks to maximize it. An adversarial game between the generator and the discriminator thereby unfolded.

The side outputs of HED-U-Net encompass both low-level detail features and high-level semantic representations. These multi-level features provide effective supervision for GAN training, thereby enhancing model stability, accelerating convergence, and improving segmentation accuracy, particularly in edge regions. In the architecture of HEE-SegGAN, the ground truth is down-sampled to generate auxiliary labels $y_{di}$ for the discriminator, which match the spatial dimensions of the corresponding side outputs. These auxiliary labels enable gradient feedback across different feature levels during training, ensuring that the model learn the underlying data distribution more effectively and improves both stability and training efficiency. Similarly, the Canny operator is applied to extract the boundaries of the ground-truth nodule regions, which are then down-sampled to produce the auxiliary labels $y_{gi}$ for the generator. These generator-specific auxiliary labels guide HEE-SegGAN to focus more on the quality of edge segmentation during training, allowing it to predict the edges of pulmonary nodules more accurately and efficiently.

During the iterative training process of GANs, the discriminator’s evaluation and feedback guide the generator to produce higher-quality segmentation results. However, GANs also suffer from training instability and difficulties in convergence. The side outputs of HED-U-Net, when combined with auxiliary labels generated from the ground truth, facilitate the learning of the underlying data distribution while simultaneously providing gradient feedback to feature maps at multiple levels. This design effectively accelerates the convergence of the model. Therefore, the integration of GAN and HED-U-Net successfully compensates for the limitations of traditional GANs.

### 3.3. Generator

**Fig 3:**
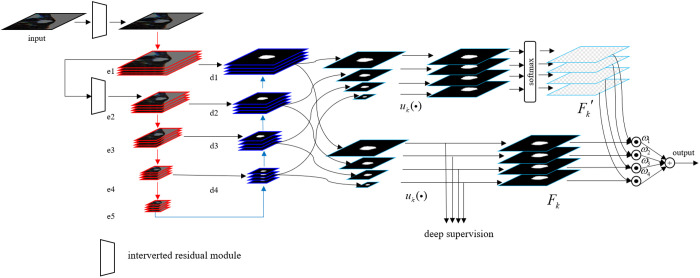
Architecture of HED-U-Net.


The network structure of HED-U-Net is shown in Fig 3. Similar to the standard U-Net, HED-U-Net consists of an encoder and a decoder. The encoder consists of four convolutional modules, each using double convolution for feature extraction and max pooling for down-sampling. The decoder mirrors this structure, restoring image features through up-sampling followed by double convolution. To further enhance multi-scale feature integration and suppress noise, a global attention mechanism is incorporated into the model. The decoder’s feature maps at different levels are denoted as $d_{k}$, (*k* = 1,2,3,4). On the feature map branch, each feature map $d_{k}$ is initially transformed into a single-channel representation via a 1 × 1 convolution $p_{k}$, and subsequently up-sampled to match the original input image size using a linear interpolation function $u_{k}$. This process yields a set of multi-scale feature maps $F_{k}$, as defined in Equation (2).

$$F_{k}=u_{k}(p_{k}(d_{k}))$$ (2)

On the parallel branch for computing attention weights, each feature map $d_{k}$ is similarly projected into a single-channel representation using a 1 × 1 convolution $q_{k}$, and subsequently up-sampled by the interpolation function $u_{k}$ to match the original input image size. After applying the softmax function to these feature maps, a set of attention weights $F\prime_{k}$ aligned with the input resolution is obtained, as defined in Equation (3).


$$F_{k}^{\prime }=soft\;\max_{k}(u_{k}(q_{k}(d_{k})))$$ (3)

The final output of HED-U-Net is defined in Equation (4).


$$\hat{y}=\sum_{k}\omega_{k}{F\prime }_{k}\cdot F_{k}$$ (4)

Where, a trainable parameter $\omega_{k}$ controls the contribution of feature maps across different scales.

**Fig 4:**
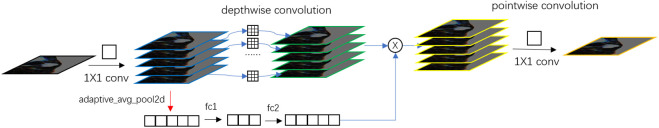
Interverted residual module.

Inspired by Mobile-NetV3 [37], two inverted residual structures are integrated into HED-U-Net to effectively fuse information from three-channel synthetic images. As illustrated in Fig 4, a 1 × 1 convolution is first applied to project the input image into a high-dimensional feature space, thereby reducing information loss. In this space, depth-wise separable convolution is employed to extract features independently from each channel. Adaptive average pooling then condenses global contextual information into a 1 × 1 vector for each feature map. These vectors are passed through two fully connected layers to generate channel-wise attention weights, which are subsequently applied to modulate their corresponding feature maps. Finally, a 1 × 1 convolution maps the refined features back to the original low-dimensional space, enhancing the spatial representation of the synthetic CT image.

### 3.4. Discriminator

To maintain the Nash equilibrium between the generator and the discriminator during HEE-SegGAN training, the discriminator is designed as a four-layer neural network. Each layer comprises a convolutional operation followed by batch normalization, a LeakyReLU activation function, and a dropout layer. A dropout rate of 20% is applied to each layer to prevent the discriminator from becoming overly dominant and to mitigate overfitting. Additionally, to preserve feature matching, the input image is concatenated with the intermediate feature maps from the discriminator to form the final output.

**Fig 5:**
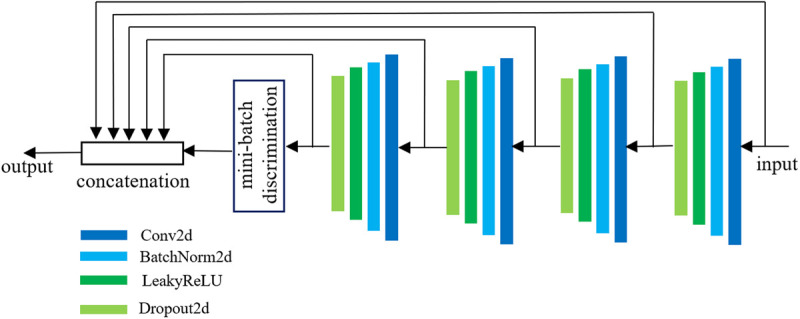
Discriminator network.


During training, all outputs within the same batch may converge to a single point that the discriminator deems highly realistic, leading to excessive similarity among samples. To address this, we introduce a mini-batch discrimination layer that penalizes overly similar samples within a batch. As illustrated in Fig 5, a batch of b samples ispassed through the four convolutional layers of the discriminator. After flattening, a feature matrix $X\in R^{b\times n}$ is obtained, where each row represents the feature vector of a single sample. Subsequently, a weight matrix W is employed to project the input features X from the original n-dimensional space to a k -dimensional space, as defined in Equation (5), yielding a transformed feature matrix $Z\in R^{b\times k}$:

Z=XW+β (5)

In Equation (5), $W\in R^{n\times k}$ denotes the weight matrix, and β represents the bias vector after feature projection. According to Equation (6), the Euclidean distance matrix $D\in R^{b\times b}$ between projected feature vectors is computed as:


$$D_{i,j}={\left\|z_{i}-z_{j}\right\|}_{2}^{2}$$ (6)

Where, $D_{i,j}$ denotes the (i,j) -th entry of matrix D, representing the squared Euclidean distance between the i -th and j -th feature vectors in the matrix Z. Specifically, $z_{i}$ and $z_{j}$ represent the feature vectors of the i -th and j -th samples, respectively, with $z_{i},z_{j}\in R^{k}$.

Subsequently, the similarity matrix $K\in R^{b\times b}$, which represents the pairwise similarity between samples, is calculated using Equation (7):


$$K_{ij}=\exp(\frac{-D_{ij}}{2\sigma^{2}})\;\;\;\;\;\;\;\;\;\;\;\;\;\;\;\;\;\;\;\;\;\sigma =k^{\frac{2}{\sqrt{n}}}$$ (7)

Here, $K_{ij}\in (0,1]$ indicates the similarity between the i -th and j -th samples in the feature space. A value of $K_{ij}\approx 1$ suggests a high degree of similarity, whereas $K_{ij}\approx 0$ implies substantial dissimilarity. The parameter σ represents the bandwidth of the Gaussian kernel and controls the sensitivity of the similarity function.

The last step is feature aggregation, where the mean similarity of each sample to all other samples is calculated:


$$M_{i}=\frac{1}{b}\sum_{j=1}^{b}K_{ij}$$ (8)

$M_{i}$ represents the average similarity of the i -th sample, reflecting its mean similarity to all other samples in the mini-batch. Finally, a similarity vector $M=[M_{1},M_{2},\;\;\;\;\ldots ,\;\;\;\;M_{b}]\in R^{b}$ is then constructed to characterize the overall similarity relationships within the mini-batch, which serves to determine whether the sample distribution is overly homogeneous.

### 3.5. Loss function

Since pulmonary nodules occupy only a small portion of the CT images, a self-balanced cross-entropy loss function is employed to compute the loss of feature maps at different levels, providing guidance for the discriminator’s decision-making. Given that the number of positive samples in the ground truth is $N_{pos}$ and the number of negative samples is $N_{neg}$, the weight of positive samples at the k -th level of the input image is defined as in Equation (9).


$$\omega_{k}^{p}=\left\{ \;\;\;\begin{array}{c} N_{neg}/N_{pos}\;\;\;\;\;\;\;\;\;\;\;\;\;\;\;\;\;\;N_{pos}>0\\ 1\;\;\;\;\;\;\;\;\;\;\;\;\;\;\;\;\;\;\;\;\;\;\;\;\;\;\;\;\;\;\;\;\;\;\;\;\;\;\;\;\;\;\;\;\;\;\;\;\;\;\;\;\;\;\;\;\;\;\;\;\;\;\;\;\;\;\;\;\;\;\;N_{pos}=0 \end{array}\right.$$ (9)

The weighted binary cross entropy loss function of the *k*-th feature map is defined in Equation (10):


$$L_{dk}=-\sum_{i}\left[\omega_{k}^{p}\cdot y_{dk}^{i}\log\sigma (F_{k}^{i})+(1-y_{dk}^{i})\log(1-\sigma (F_{k}^{i}))\right]$$ (10)

In Equation (10), σ denotes the sigmoid function, $F_{k}^{i}$ represents the value of the *i*-th pixel of the *k*-th feature map, and $y_{dk}^{i}$ denotes the value of the i -th pixel in the k-th auxiliary label for discriminator. The aggregated loss is computed as the sum of the loss functions across different levels of the CT image feature maps. The aggregated loss supervises the training of the GAN and is formulated in Equation (11).


$$L_{ds}=\sum_{k=1}^{4}L_{dk}$$ (11)

The adversarial loss function $L_{a}$ is defined in Equation (1). Consequently, the loss function of the discriminator is represented as $L_{d}$:


$$L_{d}=-L_{a}-L_{ds}$$ (12)

To address the challenge of accurately segmenting the edges of pulmonary nodules, auxiliary labels are introduced into the generator to explicitly supervise the learning of multi-level edge features. The edge-aware loss function for feature maps at different depths, designed to enhance edge localization, is defined as Equation (13):


$$L_{ge}=\sum_{k=1}^{4}L_{gk}=-\sum_{k=1}^{4}\sum_{i}\left[\omega_{k}^{p}\cdot y_{gk}^{i}\log\sigma (F_{k}^{i})+(1-y_{gk}^{i})\log(1-\sigma (F_{k}^{i}))\right]$$ (13)

The Dice loss between the generator’s final segmentation output $\hat{y}$ and the ground truth y is denoted as $L_{gs}$.


$$L_{gs}=1-2\sum y\hat{y}/(\sum y+\sum \hat{y})$$ (14)

The final loss function of the generator is defined as Equation (15), where $\omega_{e}$, $\omega_{s}$ are weight coefficients.


$$L_{g}=L_{a}+\omega_{e}L_{ge}+\omega_{s}L_{gs}$$ (15)

## 4. Experiment and result

### 4.1. Implementation details

Our model was trained on an AMAX XP-48201G deep learning server equipped with eight NVIDIA 2080Ti GPUs, each with 12 GB of VRAM. We used subsets 0–8 of the LUNA16 dataset for training and subset 9 for validation, resulting in 6,747 training samples and 736 test samples. Training was conducted until the loss function converges. A batch size of 64 was used, and both the generator and discriminator were optimized using the Adam optimizer with an initial learning rate of 0.001. The first and second-order momentum parameters were set to 0.9 and 0.999, respectively. To improve model generalization, a cosine annealing learning rate scheduler was employed to gradually decrease the learning rate. According to the Lipschitz theorem, GAN convergence requires the discriminator to satisfy Lipschitz continuity. To enforce this, we clipped the discriminator’s parameters within the range [−0.012, 0.012], which was found to stabilize training.

In both the ground truth y and the predicted mask $\hat{y}$ of the CT images, a pixel value of 1 indicates that the pixel belongs to a nodule, whereas a value of 0 denotes normal tissue. The evaluation metrics include Intersection over Union (IoU), Dice Similarity Coefficient (DSC), Sensitivity (SEN), and Precision (PRE). Table 1 defines these metrics and their significance.

**Table 1:** Evaluation Metric and significance of pulmonary nodule segmentation.

Metric	Mathematical Definition:	Meaning:
**IoU**	$\sum \hat{y}y/(\sum \hat{y}+\sum y-\sum y\hat{y})$	IoU quantifies the degree of overlap between the predicted region and the ground truth.
**DSC**	$2\sum y\hat{y}/(\sum \hat{y}+\sum y)$	DSC measures the similarity between the predicted and ground truth regions.
**SEN**	$\sum \hat{y}y/\sum y$	Sensitivity (Recall) quantifies the model’s ability to accurately identify positive regions.
**PRE**	$\sum \hat{y}y/\sum \hat{y}$	Precision measures how many predicted positive pixels are truly positive.

### 4.2. Comparison with existing algorithms

We compared our proposed method with representative approaches from the literature, including various improved U-Net models, Mask R-CNN, multi-feature fusion CNNs, and improved GANs. The comparison results, presented in Table 2, demonstrated that our method achieved superior overall performance.

**Table 2:** Comparison of our method with existing typical methods.

method	Dataset	IoU	DSC	SEN	PRE
U-Net [13]	LIDC-IDRI		0.8300	0.8980	0.7920
Seg-U-Net [13]	LIDC-IDRI		0.823	0.858	0.787
DA-Net [15]	LIDC-IDRI	0.7160	0.8100	0.8720	
DENSE-UNET [16]	LIDC-IDRI		0.7442	0.7254	0.7551
Dilated U-Net [29]	LIDC-IDRI	0.6824	0.7401		0.8609
Improved U-Net [26]	LUNA16		0.8282	0.9224	
DB U-Net [12]	LIDC-IDRI		0.8316	0.8851	
Improved V-Net [20]	LUNA16		0.6910	0.6525	0.8158
Selective kernel V-Net [18]	LUNA16	0.6650	0.7960	0.7890	
Dual-branch feature fusion S3D V-Net [21]	LUNA16		0.8457		
Mask R-CNN [6]	LUNA16		0.8290	0.8540	0.8060
Multi-feature fusion CNN [27]	LUNA16	0.7170	0.8350	0.8650	
Dual-task region-boundary aware CNN [30]	LUNA16	0.5728	0.7161	0.7189	
SSSOA-based GAN [38]	LIDC-IDRI	0.6896	0.7986		
CSE-GAN [4]	LUNA16	0.7252	0.8074	0.8546	0.8056
Our Method	LUNA16	0.7430	0.8528	0.8297	0.8770

### 4.3. Ablation experiment

An ablation study was conducted to evaluate the effectiveness of the proposed mechanisms in our model, and the corresponding results were presented in Table 3. To assess whether synthetic images better utilize the spatial information of CT scans, we performed experiments using U-Net, HED-U-Net, and GAN with mini-batch discrimination. Each model was tested with both single-channel original CT images and three-channel synthetic CT images. The results demonstrated that, when using three-channel synthetic CT images, the DSC scores of U-Net, HED-U-Net, and GAN were higher than those obtained with single-channel CT images, confirming the effectiveness of the proposed method.

**Table 3:** Results of ablation experiments.

Method	IoU	DSC	SEN	PRE
UNet(1 channel)	0.6488	0.7772	0.7984	0.7810
UNet(3 channel)	0.7272	0.8315	0.8190	0.8746
HED-UNet(1 channel)	0.6609	0.7911	0.7905	0.8160
HED-UNet(3 channel)	0.7364	0.8385	0.8519	0.8455
GAN(HED-Unet_3 channel)	0.7220	0.8357	0.8244	0.8535
GAN(HED-Unet_1channel_MB)	0.6444	0.7814	0.7555	0.8179
GAN(HED-Unet_3channel_MB)	0.7387	0.8463	0.8112	0.8959
GAN(HED-Unet_3channel_MB_IR)	0.7430	0.8528	0.8297	0.8770

Compared to U-Net, HED-U-Net employed hierarchical outputs to better utilize multi-scale features. To verify the mechanism, we trained HED-U-Net and U-Net using single-channel and three-channel synthetic CT images, respectively. The results showed that HED-U-Net outperformed U-Net by 0.0121 in DSC on single-channel CT images, and by 0.0092 on three-channel synthetic color CT images.

To assess the effectiveness of adversarial training, we used HED-U-Net as a GAN generator to form HEE-SegGAN, with segmentation results denoted as GAN (HED-U-Net_3 channel). As shown in Table 3, this approach achieved IoU = 0.7220, DSC = 0.8357, SEN = 0.8244, and PRE = 0.8535, slightly lower than those of HED-U-Net alone. The decline was primarily attributed to the GAN training process, wherein batch-wise gradient similarity reduced intra-batch diversity.

To mitigate the adverse effects of gradient convergence within the same batch, a mini-batch discrimination mechanism was integrated into the GAN framework, resulting in the variant GAN (HED-U-Net_3channel_MB). As shown in Table 3, the enhanced model achieved superior results, with an IoU of 0.7387, DSC of 0.8463, SEN of 0.8112, and PRE of 0.8959, surpassing the performance of HED-U-Net alone.

To further enhance the spatial feature extraction of three-channel synthetic images, two inverted residual modules were introduced into the GAN generator based on the mini-batch discrimination mechanism. This variant, referred to as GAN (HED-Unet_3channel_MB_IR) in Table 3, achieved the highest segmentation accuracy. Experimental results showed that this improvement yielded an IoU of 0.7430, DSC of 0. 8528, SEN of 0.8297, and PRE of 0.8770.

To evaluate the statistical significance of performance differences among algorithms and to eliminate the influence of random factors, a two-sample t-test was conducted. In the experiments, three-channel synthetic color CT images were used as the dataset. The models included U-Net, HED U-Net, GAN, GAN with mini-batch discrimination mechanism (GAN-MB), and GAN-MB with inverted residual modules (GAN-MB-IR). Each model was trained independently ten times.

**Fig 6:**
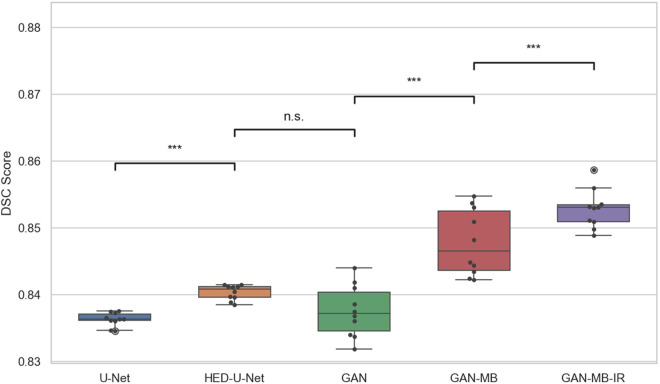
Box plot of segmentation results of different models.

The boxplots of DSC values across ten test runs for each of the five models were presented in Fig 6, illustrating the distribution and variability of segmentation performance. Pairwise comparisons between adjacent models were performed using independent sample t-tests. In the figure, statistical significance is denoted as follows: *** indicates a very significant difference with p < 0.001; ** denotes a significant difference with p < 0.01; * represents a moderate difference with p < 0.05; and n.s. indicates no statistically significant difference between the compared groups.

As shown in Fig 6, the segmentation accuracy of U-Net and HED-U-Net was relatively low, although their results exhibited less variance compared to GAN-based methods. The introduction of an edge detection mechanism in HED-U-Net led to improved segmentation accuracy over U-Net, and the statistical difference between the two was found to be highly significant. Although the segmentation performance of GAN was slightly lower than that of HED-U-Net, the difference between them was not statistically significant. With the incorporation of a mini-batch discrimination mechanism, the segmentation results of GAN-MB showed a very significant improvement over those of the original GAN. Furthermore, after integrating inverted residual modules, GAN-MB-IR achieved another highly significant performance gain.

Another ablation study was conducted to investigate the impact of different loss function combinations on segmentation performance. The GAN-MB-IR model was tested ten times using three-channel synthetic color CT images, and the corresponding results are presented in Table 4. When only the adversarial loss $L_{a}$ and Dice loss $L_{gs}$ were employed, the model achieved a DSC score of 0.8440, with an average training time of 4.33 seconds per epoch. After incorporating the supervised loss $L_{ds}$ into the discriminator, the DSC score increased to 0.8493. Due to the introduction of the supervision mechanism, the training speed improved significantly, with the average epoch duration reduced to only 1.18 seconds. Further integrating the edge loss $L_{ge}$ into the generator led to a DSC score of 0.8528.

**Table 4:** The impact of different loss function modules on segmentation results.

$L_{a}$	$L_{ds}$	$L_{gs}$	$L_{ge}$	IoU	DSC	SEN	PRE	Epoch Duration
✓		✓		0.7381	0.8440	0.8658	0.8376	4.33s
✓	✓	✓		0.7397	0.8478	0.8634	0.8385	1.18s
✓	✓	✓	✓	0.7430	0.8528	0.8297	0.8770	1.20s

**Fig 7:**
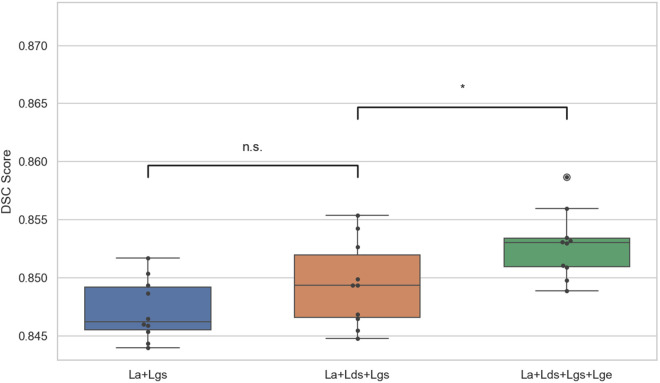
Box plot of segmentation results for different loss function combinations.

The segmentation results under different combinations of loss functions were subjected to a t-test, as shown in Fig 7. The results indicated that incorporating the supervision loss into the discriminator did not lead to a significant improvement in segmentation accuracy, although it did shorten the training time. When the edge loss was further introduced into the generator, a better balance between the generator and discriminator was achieved, resulting in an improvement in segmentation performance. Compared to the model without edge loss in the generator, the inclusion of this component led to a moderate difference in the segmentation results.

**Fig 8:**
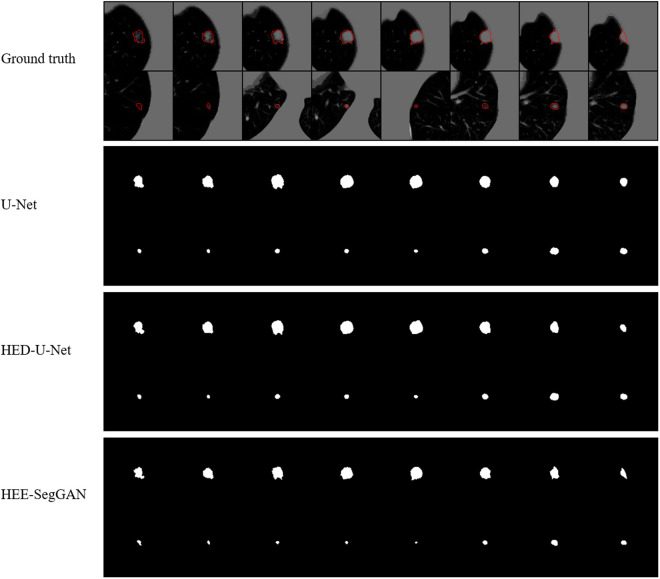
Visual effects of segmentation of different models.

We compared the segmentation performance of U-Net, HED-U-Net, and HEE-SegGAN against the ground truth. The ground truth is depicted by the red solid line in Fig 8. Compared to U-Net, HED-U-Net integrates hierarchical output and deep supervision, effectively leveraging multi-scale information to enhance lung nodule edge details. Adversarial training in HEE-SegGAN further enhanced segmentation accuracy, particularly along object boundaries.

## 5. Discussion

By comparing the existing literature in Table 2, we can find that Wu et al. [12] used an improved U-Net that combined 2D and 3D to achieve a DSC of 0.8316 on the LIDC-IDRI dataset. Xu et al. [21] used an improved V-Net that combines 2D and 3D to achieve a DSC of 0.8457 on the LUNA16 dataset. The performance of these two methods is better than other 2D methods that increase connections or introduce multi-scale mechanisms. It shows the importance of utilizing the spatial information contained in CT images. The image preprocessing method proposed in this work combined three consecutive slices into a single color image, preserving spatial information while avoiding the introduction of redundant parameters. Ablation experiments also proved the effectiveness of our method. To validate the effectiveness of the proposed preprocessing method, we conducted ablation experiments using three different models to segment pulmonary nodules in both single-slice CT images and synthesized color CT images. The results show that incorporating synthetic CT images increased the DSC by 0.0543 for UNet, 0.0474 for HED-U-Net, and 0.0649 for HEE-SegGAN. Using three-color images with spatial information improved the segmentation accuracy of all three models.

The experimental results in Table 2 also indicate that with limited data, a more complex model structure does not always necessarily yield better performance. Tang et al. [27] and Qiu et al. [30] employed a collaborative approach combining two models to enhance accuracy, achieving DSC scores of 0.8350 and 0.7161, respectively. However, their accuracy showed no significant improvement over a single model. For complex models, there is a risk of underfitting the data. Tyagi and Talbar [4] achieved a DSC of 0.7986 on the LIDC-IDRI dataset using an improved GAN, while Jain [38] obtained a DSC of 0.8074 on the LUNA16 dataset with a similar approach. However, both results remain suboptimal. The primary challenges in GAN-based medical image segmentation are maintaining training stability and ensuring that the generator accurately learns the real data distribution. As shown in Table 3, integrating the generative adversarial mechanism into HED-U-Net did not enhance segmentation accuracy but instead led to a decrease of 0.0028. To address this, we employed HED-U-Net as the generator with hierarchical supervision via side outputs and global attention mechanisms, enabling multi-scale feature integration and ensuring that the generated segmentation maps closely resemble real structures. Additionally, we introduced inverted residual modules with channel attention and depth-wise separable convolutions to improve spatial feature extraction while maintaining computational efficiency. After adding inverted residual modules, the DSC of HEE-SegGAN is improved by 0.0065. Edge delineation remains a critical issue in medical image segmentation. HEE-SegGAN mitigates this challenge by incorporating a weighted binary cross-entropy loss at multiple levels, emphasizing edge regions to improve segmentation precision. As can be seen from Fig 8, this method effectively refines edge details and outperforms the traditional U-Net and HED-U-Net models.

In the ablation study, a two-sample *t*-test was conducted to evaluate the statistical significance of segmentation performance differences across models. The results showed that although the GAN-based model achieved higher segmentation accuracy, its output stability was inferior to that of conventional architectures such as HED-U-Net and U-Net. In the experiments on loss function ablation, it was observed that introducing a supervised loss into the discriminator did not significantly improve segmentation accuracy. However, it effectively provided gradient feedback to feature maps at different levels, which greatly accelerated the convergence of the model. When an edge loss was additionally applied to the generator, calculated from auxiliary labels and side outputs of the generator, a more balanced performance was observed between the generator and the discriminator. This modification further improved the convergence behavior of the overall model. Moreover, the inclusion of edge loss in the generator enhanced the model’s caution in handling uncertain regions, due to the explicit edge supervision. The asymmetric penalty mechanism of the loss function also led to more conservative predictions. As a result, the model demonstrated a decrease in sensitivity SEN and an increase in precision PRE.

Compared with previous GAN-based methods for lung nodule segmentation, HEE-SegGAN not only achieves higher segmentation accuracy but also demonstrates better edge preservation and stability in adversarial training. However, our study has some limitations. The three-slice encoding strategy, while effective, may not fully capture complex volumetric dependencies in highly irregular nodules. Future work will explore dynamic slice selection strategies or hybrid 2.5D/3D approaches to further enhance performance. Additionally, while our method shows potential for applications in MRI, ultrasound, and PET segmentation, further studies are needed to confirm its effectiveness in these modalities.

## 6. Conclusion

In this study, we proposed HEE-SegGAN, a novel framework for pulmonary nodule segmentation that synthesizes color CT images by encoding three consecutive slices into RGB channels. This approach effectively captures inter-slice spatial dependencies while maintaining significantly lower computational costs than conventional 3D methods.

HEE-SegGAN employs HED-U-Net as the generator and a CNN-based discriminator, integrating hierarchical multi-scale supervision and global attention to enhance feature learning. Additionally, inverted residual modules were integrated to improve spatial feature extraction, and mini-batch discrimination was employed to stabilize GAN training. These innovations collectively enhance segmentation accuracy, particularly at lung nodule edges. Extensive experiments on the LUNA16 dataset demonstrate that our method outperforms single-slice-based models, achieving an IoU of 0.7430 and a DSC of 0.8528, while requiring significantly fewer parameters than 3D CNN architectures. The integration of adjacent CT slices enables the model to better differentiate nodules from surrounding lung structures, and the use of hierarchical supervision in GAN training improves stability and robustness. Furthermore, our edge-enhanced loss function significantly refines segmentation in challenging regions, addressing a key limitation in medical image analysis.

Beyond lung nodule segmentation, HEE-SegGAN holds promise for broader medical imaging applications involving layered scans, such as MRI, ultrasound, and PET. Future work will focus on optimizing network architectures and loss functions to further enhance edge segmentation performance. Additionally, we plan to explore self-supervised learning, transformer-based models, and domain adaptation techniques to improve the generalizability of our approach across diverse medical imaging datasets.

## References

[1] Nierengarten MB. Global cancer statistics 2022: The report offers a view on disparities in the incidence and mortality of cancer by sex and region worldwide and on the areas needing attention. Cancer. 2024;130(15):2568. doi: 10.1002/cncr.35444 39032060

[2] Abraham J. Reduced lung cancer mortality with low-dose computed tomographic screening. Community Oncol. 2011;8(10): 441–2. doi: 10.1056/NEJMoa1102873 PMC4356534 21714641

[3] Zhang X, Liu X, Zhang B, Dong J, Zhang B, Zhao S, et al. Accurate segmentation for different types of lung nodules on CT images using improved U-Net convolutional network. Medicine (Baltimore). 2021;100(40):e27491. doi: 10.1097/MD.0000000000027491 34622882 PMC8500581

[4] Tyagi S, Talbar SN. CSE-GAN: A 3D conditional generative adversarial network with concurrent squeeze-and-excitation blocks for lung nodule segmentation. Comput Biol Med. 2022;147:105781. doi: 10.1016/j.compbiomed.2022.105781 35777084

[5] Zhou T, Dong Y, Lu H, Zheng X, Qiu S, Hou S. APU-Net: An Attention Mechanism Parallel U-Net for Lung Tumor Segmentation. Biomed Res Int. 2022;2022:5303651. doi: 10.1155/2022/5303651 35586818 PMC9110197

[6] Wu L, Zhuang J, Chen W, Tang Y, Hou C, Li C, et al. Data augmentation based on multiple oversampling fusion for medical image segmentation. PLoS One. 2022;17(10):e0274522. doi: 10.1371/journal.pone.0274522 36256637 PMC9578635

[7] Shi HQ, Lu JG, Zhou QJ, IEEE. A Novel Data Augmentation Method Using Style-Based GAN for Robust Pulmonary Nodule Segmentation. 32nd Chinese Control And Decision Conference (CCDC). IEEE; 2020, p. 2486–91.

[8] Chen W, Wang QL, Wang K, Yang D, Zhang XH, Liu C, et al. MTGAN: Mask and Texture-driven Generative Adversarial Network for Lung Nodule Segmentation. 25th International Conference on Pattern Recognition (ICPR). IEEE; 2021, p. 1029–35.

[9] Mukherjee J, Poddar T, Kar M, Ganguli B, Chakrabarti A, Das S. An automated classification methodology of sub-centimeter pulmonary structures in computed tomography images. Comput Electric Eng. 2020;84:106629. doi: 10.1016/j.compeleceng.2020.106629

[10] Ni Y, Xie Z, Zheng D, Yang Y, Wang W. Two-stage multitask U-Net construction for pulmonary nodule segmentation and malignancy risk prediction. Quant Imaging Med Surg. 2022;12(1):292–309. doi: 10.21037/qims-21-19 34993079 PMC8666775

[11] Zhou Z, Gou F, Tan Y, Wu J. A Cascaded Multi-Stage Framework for Automatic Detection and Segmentation of Pulmonary Nodules in Developing Countries. IEEE J Biomed Health Inform. 2022;26(11):5619–30. doi: 10.1109/JBHI.2022.3198509 35984795

[12] Wu Z, Zhou Q, Wang F. Coarse-to-Fine Lung Nodule Segmentation in CT Images With Image Enhancement and Dual-Branch Network. IEEE Access. 2021;9:7255–62. doi: 10.1109/access.2021.3049379

[13] Rocha J, Cunha A, Mendonça AM. Conventional Filtering Versus U-Net Based Models for Pulmonary Nodule Segmentation in CT Images. J Med Syst. 2020;44(4):81. doi: 10.1007/s10916-020-1541-9 32140870

[14] Wang B, Chen K, Tian X, Yang Y, Zhang X. An effective deep network for automatic segmentation of complex lung tumors in CT images. Med Phys. 2021;48(9):5004–16. doi: 10.1002/mp.15074 34224147

[15] Maqsood M, Yasmin S, Mehmood I, Bukhari M, Kim M. An Efficient DA-Net Architecture for Lung Nodule Segmentation. Mathematics. 2021;9(13):1457. doi: 10.3390/math9131457

[16] Lu D, Chu J, Zhao R, Zhang Y, Tian G. A Novel Deep Learning Network and Its Application for Pulmonary Nodule Segmentation. Comput Intell Neurosci. 2022;2022:7124902. doi: 10.1155/2022/7124902 35619752 PMC9129945

[17] Qian L, Wen C, Li Y, Hu Z, Zhou X, Xia X, et al. Multi-scale context UNet-like network with redesigned skip connections for medical image segmentation. Comput Methods Programs Biomed. 2024;243:107885. doi: 10.1016/j.cmpb.2023.107885 37897988

[18] Wang Z, Men J, Zhang F. Improved V-Net lung nodule segmentation method based on selective kernel. SIViP. 2022;17(5):1763–74. doi: 10.1007/s11760-022-02387-w

[19] Kido S, Kidera S, Hirano Y, Mabu S, Kamiya T, Tanaka N, et al. Segmentation of Lung Nodules on CT Images Using a Nested Three-Dimensional Fully Connected Convolutional Network. Front Artif Intell. 2022;5:782225. doi: 10.3389/frai.2022.782225 35252849 PMC8892185

[20] Lin HB, Zhang YH, Chen XF, Wang HA, Xia LZ. Research on pulmonary nodule segmentation algorithm based on improved V-Net. 6th IEEE Advanced Information Technology, Electronic and Automation Control Conference (IEEE IAEAC). 2022, p. 194–8.

[21] Xu X, Du L, Yin D. Dual-branch feature fusion S3D V-Net network for lung nodules segmentation. J Appl Clin Med Phys. 2024;25(6):e14331. doi: 10.1002/acm2.14331 38478388 PMC11163502

[22] Zhou C, Zhao X, Zhao L, Liu J, Chen Z, Fang S. Deep Learning-Based CT Imaging in the Diagnosis of Treatment Effect of Pulmonary Nodules and Radiofrequency Ablation. Comput Intell Neurosci. 2022;2022:7326537. doi: 10.1155/2022/7326537 35996649 PMC9392615

[23] Dong L, Liu HY. Segmentation of Pulmonary Nodules Based on Improved UNet++. 14th International Congress on Image and Signal Processing, BioMedical Engineering and Informatics (CISP-BMEI). 2021, p. 1–5.

[24] Xie SN, Girshick R, Dollár P, Tu ZW, He KM. Aggregated Residual Transformations for Deep Neural Networks. 30th IEEE/CVF Conference on Computer Vision and Pattern Recognition (CVPR). 2017, p. 5987–95.

[25] Roy AG, Navab N, Wachinger C. Recalibrating Fully Convolutional Networks With Spatial and Channel “Squeeze and Excitation” Blocks. IEEE Trans Med Imaging. 2019;38(2):540–9. doi: 10.1109/TMI.2018.2867261 30716024

[26] Annavarapu CSR, Parisapogu SAB, Keetha NV, Donta PK, Rajita G. A Bi-FPN-Based Encoder-Decoder Model for Lung Nodule Image Segmentation. Diagnostics (Basel). 2023;13(8):1406. doi: 10.3390/diagnostics13081406 37189507 PMC10137204

[27] Tang T, Li F, Jiang M, Xia X, Zhang R, Lin K. Improved Complementary Pulmonary Nodule Segmentation Model Based on Multi-Feature Fusion. Entropy (Basel). 2022;24(12):1755. doi: 10.3390/e24121755 36554161 PMC9778431

[28] Zhao J, Dang M, Chen Z, Wan L. DSU-Net: Distraction-Sensitive U-Net for 3D lung tumor segmentation. Eng Appl Artific Intell. 2022;109:104649. doi: 10.1016/j.engappai.2021.104649

[29] Liu C, Pang M. Lung CT Image Segmentation via Dilated U-Net Model and Multi-scale Gray Correlation-Based Approach. Circ Syst Sig Process. 2023;43(3):1697–714. doi: 10.1007/s00034-023-02532-x

[30] Qiu J, Li B, Liao R, Mo H, Tian L. A dual-task region-boundary aware neural network for accurate pulmonary nodule segmentation. J Vis Commun Image Represent. 2023;96:103909. doi: 10.1016/j.jvcir.2023.103909

[31] Goodfellow IJ, Pouget-Abadie J, Mirza M, Xu B, Warde-Farley D, Ozair S, et al. Generative Adversarial Nets. 28th Conference on Neural Information Processing Systems (NIPS). 2014, p. 2672–80.

[32] Xue Y, Xu T, Zhang H, Long LR, Huang X. SegAN: Adversarial Network with Multi-scale L1 Loss for Medical Image Segmentation. Neuroinformatics. 2018;16(3–4):383–92. doi: 10.1007/s12021-018-9377-x 29725916 PMC13344194

[33] Xie S, Tu Z. Holistically-nested edge detection. In: Proceedings of the IEEE International Conference on Computer Vision. 2015, p. 1395–403.

[34] Heidler K, Mou L, Baumhoer C, Dietz A, Zhu XX. HED-UNet: Combined Segmentation and Edge Detection for Monitoring the Antarctic Coastline. IEEE Trans Geosci Remote Sensing. 2022;60:1–14. doi: 10.1109/tgrs.2021.3064606

[35] Setio AAA, Traverso A, de Bel T, Berens MSN, ven den Bogaard C, Cerello P, et al. Validation, comparison, and combination of algorithms for automatic detection of pulmonary nodules in computed tomography images: The LUNA16 challenge. Med Image Anal. 2017;42:1–13. doi: 10.1016/j.media.2017.06.015 28732268

[36] Hesamian MH, Jia W, He X, Wang Q, Kennedy PJ. Synthetic CT images for semi-sequential detection and segmentation of lung nodules. Appl Intell. 2020;51(3):1616–28. doi: 10.1007/s10489-020-01914-x

[37] Howard A, Sandler M, Chu G, Chen LC, Chen B, Tan MX, et al. Searching for MobileNetV3. IEEE/CVF International Conference on Computer Vision (ICCV). 2019, p. 1314–24.

[38] Jain S, Indora S, Atal DK. Lung nodule segmentation using Salp Shuffled Shepherd Optimization Algorithm-based Generative Adversarial Network. Comput Biol Med. 2021;137:104811. doi: 10.1016/j.compbiomed.2021.104811 34492518

